# Spatial and Directional Modulation Systems for Near-Field Secure Transmission

**DOI:** 10.3390/s26031065

**Published:** 2026-02-06

**Authors:** Ji Liu, Yuan Zhong, Yong Wang, Dong Gong, Yue Xiao

**Affiliations:** 1 School of Telecommunications Engineering, Xidian University, Xi’an 710126, China; 2 China Academy of Space Technology (Xi’an), Xi’an 710100, China; 3 National Key Laboratory of Wireless Communications, University of Electronic Science and Technology of China, Chengdu 611731, China

**Keywords:** spatial and directional modulation (SDM), artificial noise (AN), near-field, bit error rate (BER)

## Abstract

The proliferation of massive antenna arrays and the consequent intensification of near-field effects with 6G necessitate addressing critical security challenges in near-field communication environments. This paper presents a novel artificial noise-aided spatial and directional modulation (SDMN-AN) framework, specifically tailored for secure near-field communications. The proposed system integrates legitimate receiver indices, modulation symbols, and artificial noise (AN) confined to the null space of legitimate channels, thereby enhancing both spectral efficiency and communication security. Two precoding strategies—maximum-ratio transmission (MRT) and zero-forcing (ZF)—are investigated, offering trade-offs between hardware complexity and detection overhead. Analytical derivations of bit error rate (BER) bounds, corroborated by simulation results, underscore the superiority of the SDMN-AN framework in mitigating eavesdropping threats while significantly improving spectral efficiency, positioning it as a compelling solution for next-generation secure wireless networks.

## 1. Introduction

The vision of sixth-generation (6G) wireless communications [1,2,3] heralds an era of truly pervasive and intelligent connectivity, where massive numbers of heterogeneous devices, sensors, and users are seamlessly integrated into a unified cyber–physical–digital continuum. Enabled by disruptive physical-layer technologies such as extremely large-scale multiple-input multiple-output (XL-MIMO), 6G networks fundamentally reshape conventional propagation assumptions by operating deep into the electromagnetic near-field regime [4,5,6]. This paradigm shift profoundly alters the spatial characteristics of wireless channels and, in turn, the design philosophy of communication systems.

While such unprecedented connectivity promises transformative gains in spectral efficiency, reliability, and service diversity, it simultaneously amplifies the exposure of sensitive information to sophisticated eavesdropping threats inherent in densely connected and highly dynamic 6G environments [7]. Consequently, ensuring information confidentiality can no longer rely solely on traditional security mechanisms. Instead, there is a compelling need to develop robust and intrinsically secure transmission strategies that explicitly account for the unique challenges posed by near-field propagation in MIMO-enabled 6G systems [8].

Although significant advancements have been realized in the domain of far-field physical-layer secure communications, these solutions face fundamental limitations when extrapolated to the near-field regime. In far-field settings, directional modulation (DM) represents a cornerstone innovation, leveraging sophisticated beamforming techniques to confine signal energy within narrowly defined spatial regions [9]. This targeted approach mitigated interception risks, as demonstrated in studies such as [10,11], which illustrated efficacy of DM in multi-user environments. Furthermore, efficiency-optimized designs, exemplified by the work in [12], have underscored the critical role of phase shifter sequence engineering in augmenting security. Nevertheless, these methodologies presuppose planar wavefront characteristics, inherently constraining their utility to far-field conditions.

Augmenting DM techniques, artificial noise (AN) has emerged as a robust adjunct for far-field secure communication systems [13]. AN disrupts eavesdroppers by introducing structured interference into null spaces of legitimate channels, thereby preserving the performance of authorized users. Research by [14] highlighted the effectiveness of AN in thwarting eavesdropping attempts, while the work of [15] has explored its integration with DM to bolster security. Furthermore, the authors of [16] extended these frameworks through the joint incorporation of spatial and directional modulation alongside AN, achieving significant security and spectral efficiency gains under realistic channel conditions. However, such approaches remain intrinsically bounded by far-field approximations, rendering them inapplicable to the complex propagation characteristics of the near-field regime.

The deployment of ultra-large-scale antenna arrays introduces pronounced near-field effects, characterized by spherical wavefront propagation [17]. These effects necessitate novel security paradigms, as traditional far-field algorithms fail to adequately address the challenges posed by near-field environments. Initial research efforts have sought to adapt DM and AN to exploit near-field spatial characteristics, achieving enhanced selectivity and robust eavesdropping resilience. For instance, the study in [18] investigated the potential of near-field hybrid beamforming for secure communications, while the authors of ref. [19] demonstrated the efficacy and security of combined DM-AN techniques in short-range scenarios. However, existing methodologies predominantly target point-to-point scenarios, neglecting the potential of multi-point collaboration and often experiencing degraded security when eavesdropping receivers operate in close proximity of legitimate receivers.

To address these gaps, this paper proposes a novel artificial noise-aided spatial and directional modulation for near-field (SDMN-AN) system, specifically applied for secure communications within near-field contexts. Unlike conventional far-field schemes, the proposed design explicitly exploits the angle–distance coupling induced by near-field spherical wavefronts, enabling distance-dependent spatial discrimination and receiver-index modulation (IM) that are infeasible under plane-wave assumptions. This near-field property fundamentally enhances AN effectiveness and secure signal separation even when legitimate and illegitimate receivers are closely aligned in angle. The proposed SDMN-AN framework is particularly applicable to cooperative sensor networks and indoor short-range sensing systems, where near-field propagation and distributed receivers are inherent characteristics.

The primary contributions of this work are delineated as follows:

- Multi-point collaborative architecture: The proposed SDMN-AN framework integrates a multi-point cooperative receiver strategy at the legitimate user, Bob, while the transmitter, Alice, employs a combination of receiver indices and modulation symbols to significantly enhance spectral efficiency. By explicitly operating in the near-field regime, the proposed architecture exploits the inherent angle–distance coupling of spherical wavefronts, which enables distance-dependent spatial discrimination among distributed receivers. The inclusion of AN further disrupts the adversarial eavesdropper Eve’s signal reconstruction capabilities due to near-field-induced null-space mismatch, thereby ensuring robust physical-layer security even when Eve is closely aligned with Bob in angle under hostile conditions.
- Dual precoding and detection strategies: Two distinct configurations are examined—(i) maximum-ratio transmission (MRT) precoding paired with joint maximum-likelihood (ML) detection, optimized for minimal hardware complexity at the transmitter, and (ii) zero-forcing (ZF) precoding coupled with joint ML detection, aimed at alleviating computational demands at the receiver.
- Analytical and empirical validation: Closed-form expressions for the bit error rate (BER) upper bounds of both the legitimate receiver and the eavesdropper are derived. Simulation results confirm superior performance of the proposed system in achieving multi-point cooperative security gains for near-field regions, significantly surpassing the security and communication efficiency of existing DM-AN systems.

The structure of this paper is organized as follows: Section 2 and Section 3 delineate the system model for the proposed SDMN-AN framework, detailing its two distinct precoding schemes. Section 4 rigorously derives the upper bounds of the BER for both Bob and Eve. Section 5 provides a comprehensive performance evaluation, supported by simulation results and analytical insights. Finally, Section 6 encapsulates the primary findings and offers concluding remarks.

Notation: In this paper, bold lowercase and uppercase symbols are used to represent column vectors and matrices, respectively, while the transpose and conjugate transpose operators are denoted by ${\left(\cdot \right)}^{T}$ and ${\left(\cdot \right)}^{H}$. The Frobenius norm and the real part of a scalar are expressed as $\left|\cdot \right|$ and R(·), respectively. Furthermore, CN(·) denotes the circularly symmetric complex Gaussian distribution, while Q(·) represents the Gaussian *Q*-function. The identity matrix of dimension *N* is symbolized by $I_{N}$.

## 2. System Model

**Figure 1:**
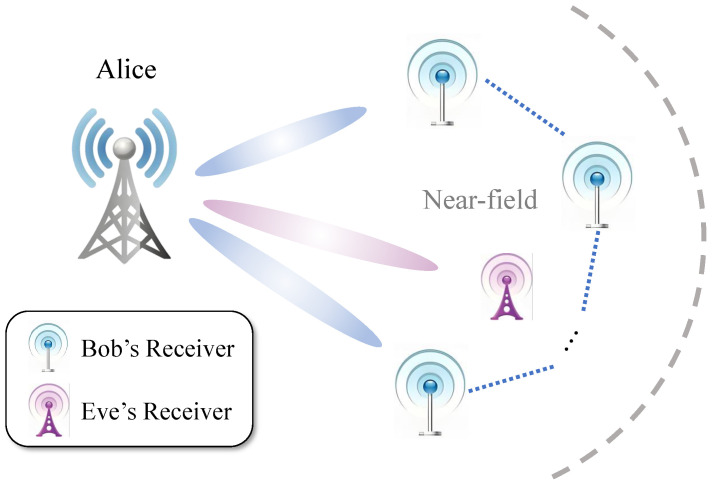
The SDMN-AN model.

As illustrated in Figure 1, we consider a downlink transmission scenario in which the transmitter, referred to as Alice, is equipped with $N_{t}$ antennas arranged in a uniform linear array (ULA). The intended receiver, Bob, employs $N_{r}=2^{\xi_{1}}$ spatially distributed single-antenna nodes, whereas the passive eavesdropper, Eve, is equipped with $N_{e}$ distributed single-antenna receivers. Notably, both Bob’s and Eve’s receivers operate within Alice’s near-field propagation region. Additionally, the distributed receivers at Bob and Eve are interconnected via optical fiber links, enabling inter-receiver communication within their respective domains.

**Figure 2:**
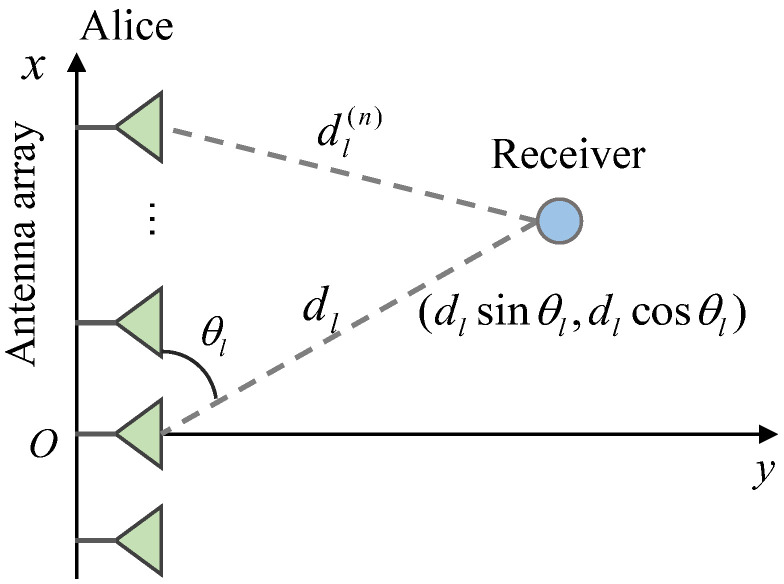
The near-field model in terms of the SDMN-AN system.

Referring to Figure 2, the position of the *n*-th antenna at Alice is characterized by the coordinates $\left(\delta_{n}D,0\right)$, where $\delta_{n}=n-1-\frac{N_{t}-1}{2}$. Considering the steering angle $\theta_{l}$ and the distance $d_{l}$ of the *l*-th receiver relative to the center of Alice’s ULA, the coordinates of the *l*-th receiver are given by $\left(d_{l}\;\cos\theta_{l},d_{l}\;\sin\theta_{l}\right)$.

In practical near-field environments, the Alice-to-receiver link typically exhibits a mixed LoS/NLoS propagation structure. Accordingly, the near-field channel vector $h_{l}\in C^{N_{t}}$ between Alice and the *l*-th receiver is modeled as a superposition of a deterministic LoS component and a scattered NLoS component, i.e.,

(1) $$h_{l}=\sqrt{\frac{\kappa_{l}}{1+\kappa_{l}}}\;g_{l,0}\left(d_{l,0}\right)\;\beta \left(\theta_{l,0},d_{l,0}\right)+\sqrt{\frac{1}{1+\kappa_{l}}}\sum_{p=1}^{L_{l}}\alpha_{l,p}\;g_{l,p}\left(d_{l,p}\right)\;\beta \left(\theta_{l,p},d_{l,p}\right),$$

where $\kappa_{l}$ denotes the Rician factor controlling the power ratio between the LoS and NLoS components, $\left(\theta_{l,0},d_{l,0}\right)$ represents the angle and distance of the LoS path, and ${\left\{\left(\theta_{l,p},d_{l,p}\right)\right\}}_{p=1}^{L_{l}}$ characterize the $L_{l}$ NLoS paths. Moreover, $\alpha_{l,p}$ denotes the complex gain of the *p*-th NLoS path, and $g_{l,p}\left(d_{l,p}\right)=\frac{\lambda_{c}}{4\pi d_{l,p}}$ is the corresponding path-loss coefficient with $\lambda_{c}$ being the carrier wavelength.

The near-field steering vector $\beta \left(\theta_{l,p},d_{l,p}\right)\in C^{N_{t}}$ associated with the *p*-th propagation path of the *l*-th receiver is given by

(2) $$\beta \left(\theta_{l,p},d_{l,p}\right)=\frac{1}{\sqrt{N_{t}}}{\left[e^{-j\frac{2\pi }{\lambda_{c}}\left(d_{l,p}^{\left(0\right)}-d_{l,p}\right)},\ldots ,e^{-j\frac{2\pi }{\lambda_{c}}\left(d_{l,p}^{\left(N_{t}-1\right)}-d_{l,p}\right)}\right]}^{T},$$

where $d_{l,p}^{\left(n\right)}$ denotes the propagation distance between the *n*-th transmit antenna and the *l*-th receiver along the *p*-th path. According to the geometric relationship illustrated in Figure 2, $d_{l,p}^{\left(n\right)}$ can be expressed as

(3) $$d_{l,p}^{\left(n\right)}=\sqrt{d_{l,p}^{2}\sin^{2}\theta_{l,p}+{\left(d_{l,p}\cos\theta_{l,p}-\delta_{n}D\right)}^{2}}=\sqrt{d_{l,p}^{2}+\delta_{n}^{2}D^{2}-2d_{l,p}\delta_{n}D\cos\theta_{l,p}},$$

with $\delta_{n}=n-1-\frac{N_{t}-1}{2}$. Unlike conventional far-field planar-wave models, the spherical-wave formulation in (1)–(3) introduces distance-dependent phase variations across the transmit array, which break the shift-invariant channel structure and alter spatial orthogonality. As a result, receivers with similar angles but different distances exhibit distinct channel responses in the near-field regime, enabling distance-selective transmission and enhanced physical-layer security.

Although a mixed LoS/NLoS near-field propagation model is considered, we focus on an LoS-dominant scenario, which is commonly encountered in short-range near-field communications. In this case, the overall channel structure is primarily governed by the direct LoS path, and thus the channel can be effectively parameterized by the geometric parameters of the LoS component. Assuming that the receivers at Bob are located at $\left(\theta_{l}^{b},d_{l}^{b}\right)$ for $l=1,\;2,\;\ldots ,\;N_{r}$, the channel matrix $H_{b}\in C^{N_{r}\times N_{t}}$ between Alice and Bob accounting for all receivers at Bob is cast as

(4) $$H_{b}={\left[h\left(\theta_{1}^{b},d_{1}^{b}\right),\;h\left(\theta_{2}^{b},d_{2}^{b}\right),\;\ldots ,\;h\left(\theta_{N_{r}}^{b},d_{N_{r}}^{b}\right)\right]}^{H}.$$

Similarly, assuming that the receivers at Eve are positioned at $\left(\theta_{l}^{e},d_{l}^{e}\right)$ for $l=1,\;2,\;\ldots ,\;N_{e}$, the channel matrix $H_{e}\in C^{N_{e}\times N_{t}}$ between Alice and Eve considering all receivers at Eve is represented as

(5) $$H_{e}={\left[h\left(\theta_{1}^{e},d_{1}^{e}\right),\;h\left(\theta_{2}^{e},d_{2}^{e}\right),\;\ldots ,\;h\left(\theta_{N_{e}}^{e},d_{N_{e}}^{e}\right)\right]}^{H}.$$

## 3. Proposed Transmission Design of Near-Field SDMN-AN

The core concept of SDMN-AN is to exploit both the indices of distributed receivers and traditional *M*-ary amplitude–phase modulation (APM) symbols with $M=2^{\xi_{2}}$, this design allows part of the information bits to be conveyed through receiver indices rather than constellation symbols. In total, $\xi =\xi_{1}+\xi_{2}$ bits are transmitted during each time slot, thereby enhancing spectral efficiency. These ξ bits are organized into an SDMN-AN super symbol, denoted as $x_{j}^{m}$ for $1\leq j\leq N_{r}$ and 1≤m≤M, which is expressed as

(6) $$x_{j}^{m}=v_{j}\alpha_{m},$$

where $v_{j}$ is the *j*-th column of $I_{N_{r}}$ and $\alpha_{m}\in A=\left\{\alpha_{1},\alpha_{2},\ldots ,\alpha_{M}\right\}$ denotes a traditional APM symbol.

Furthermore, near-field channel estimation for the Alice-Bob link is performed by Alice through the application of a specific transformation matrix to generate the precoder matrix. To leverage the intrinsic sparsity of near-field channels, a polar-domain transformation was proposed in [20], where a dedicated transformation matrix, denoted by Ψ, is employed to project the near-field channel H into the polar domain. The resulting transformation matrix is given by

(7) $$\begin{array}{cc} \Psi = & \left[\psi \left(\theta_{1},r_{1}^{1}\right),\ldots ,\psi \left(\theta_{1},r_{1}^{S_{1}}\right),\ldots ,\right.\\ \; & \left.\psi \left(\theta_{N_{1}},r_{N_{1}}^{1}\right),\ldots ,\psi \left(\theta_{N_{1}},r_{N_{1}}^{S_{N_{1}}}\right)\right], \end{array}$$

Each column of the polar-domain transformation matrix Ψ corresponds to a near-field array response vector evaluated at a specific polar grid point $\left(\theta_{n_{1}},r_{n_{1}}^{S_{n_{1}}}\right)$, where $S_{n_{1}}=1,2,\ldots ,S_{N_{1}}$. Here, $S_{N_{1}}$ denotes the number of discretized distance samples associated with the angular grid $\theta_{n_{1}}$. Consequently, the total number of polar sampling points that jointly span the entire propagation region can be expressed as

(8) $$S=\sum_{n_{1}=1}^{N_{1}}S_{N_{1}}.$$

Using the transformation matrix Ψ, the channel $H_{b}$ can be approximated as $H_{b}\approx {\bar{H}}_{b}={\left(\Psi H_{b}^{P}\right)}^{H}$, where $H_{b}^{P}\in C^{S\times N_{r}}$ represents the sparse polar-domain channel representation, obtained through the corresponding polar-domain compressed sensing algorithm. Based on this approximation, the precoder matrix and associated detection mechanisms can be derived from $H_{b}$. However, due to the constraints on hardware overhead and detection complexity at Alice and Bob, two distinct transmission and detection schemes are considered.

### 3.1. MRT Precoding and Joint ML Detection

To mitigate the hardware overhead at Alice, the proposed scheme employs MRT precoding. The precoding matrix is expressed as

(9) $$W_{M}={{\bar{H}}_{b}}^{H}.$$

where the matrix $W_{M}$ operates in the analog domain, thereby significantly reducing hardware requirements and power consumption. To enhance transmission security, especially in scenarios where Eve is in close vicinity to Bob, AN is injected into the null space of ${\bar{H}}_{b}$. To this end, the singular value decomposition (SVD) of ${\bar{H}}_{b}$ is given by

(10) $${\bar{H}}_{b}=U[\Sigma \;0][V\;V_{N}{]}^{H},$$

where $U\in C^{N_{r}\times N_{r}}$ and $V\in C^{N_{t}\times N_{r}}$ denote the left and right singular vector matrices, respectively. The diagonal matrix $\Sigma =\mathrm{diag}(\sigma_{1},\sigma_{2},\ldots ,\sigma_{N_{r}})$ collects the nonzero singular values of ${\bar{H}}_{b}$, while $V_{N}\in C^{N_{t}\times \left(N_{t}-N_{r}\right)}$ spans the null space of ${\bar{H}}_{b}$, satisfying ${\bar{H}}_{b}V_{N}=0$. It is worth noting that this orthogonality property generally no longer holds when Eve is located at a position different from Bob, i.e., ${\bar{H}}_{e}V_{N}\neq 0$.

Accordingly, the transmitted signal can be expressed as

(11) $$s_{j}^{m}=\rho \;W_{M}x_{j}^{m}+V_{N}n_{0},$$

where ρ denotes the power scaling factor and $n_{0}\in C^{\left(N_{t}-N_{r}\right)}$ represents the AN vector whose entries are zero-mean random variables with variance $\sigma_{r}^{2}$. By defining γ as the proportion of transmit power allocated to $W_{M}$, the corresponding expressions for ρ and $\sigma_{r}^{2}$ can be readily obtained as

(12) $$\rho =\sqrt{\frac{\gamma N_{r}}{\mathrm{tr}(WMW_{M}^{H})}},$$

and

(13) $$\sigma_{r}^{2}=\frac{\left(1-\gamma \right)N_{r}}{N_{t}-N_{r}}.$$

For Bob, the received signal is given by

(14) $$\begin{array}{cc} y_{b} & =H_{b}s_{j}^{m}+n_{b}\\ \; & =H_{b}\left(\rho W_{M}x_{j}^{m}+V_{N}n_{0}\right)+n_{b}\\ \; & =\rho H_{b}W_{M}x_{j}^{m}+n_{b}. \end{array}$$

For Eve, the received signal is expressed as

(15) $$\begin{array}{cc} y_{e} & =H_{e}s_{j}^{m}+n_{e}\\ \; & =H_{e}\left(\rho W_{M}x_{j}^{m}+V_{N}n_{0}\right)+n_{e}\\ \; & =\rho H_{e}W_{M}x_{j}^{m}+H_{e}V_{N}n_{0}+n_{e}. \end{array}$$

where $n_{b}∼𝒞𝒩\left(0,\sigma_{b}^{2}I_{N_{r}}\right)$ and $n_{e}∼𝒞𝒩\left(0,\sigma_{e}^{2}I_{N_{e}}\right)$ represent complex Gaussian noise vectors at Bob and Eve, respectively. Assuming that $N_{r}=N_{e}$, when the joint ML detector is employed, Bob’s detection process can be represented as

(16) $$\left\{\hat{j},{\hat{\alpha }}_{m}\right\}=\underset{1\leq j\leq N_{r},\alpha_{m}\in A}{\arg\min}{∥y_{b}-\rho {\bar{H}}_{b}W_{M}x_{j}^{m}∥}^{2}.$$

Similarly, the detection procedure at Eve can be expressed as

(17) $$\left\{\hat{j},{\hat{\alpha }}_{m}\right\}=\underset{1\leq j\leq N_{r},\alpha_{m}\in A}{\arg\min}{∥y_{e}-\rho {\bar{H}}_{e}W_{M}x_{j}^{m}∥}^{2}.$$

In particular, from Equation (14), it is evident that AN has no effect on Bob’s received signal. In contrast, the terms $\rho H_{e}W_{M}$ and $H_{e}V_{N}n_{0}$ in Equation (15) both distort the transmitted signal in terms of both amplitude and phase, resulting in a highly degraded signal at Eve. Even though Eve has accurately estimated its near-field channel, the presence of the AN precludes it from reliably recovering the transmitted signal. Thus, this approach reduces Alice’s hardware overhead while simultaneously ensuring robust security performance and considered spectral efficiency. However, the detection complexity at Bob cannot be neglected, and a solution to alleviate this detection complexity is discussed in the subsequent section.

### 3.2. ZF Precoding and Joint ML Detection

The proposed scheme employs a ZF precoding matrix to mitigate the detection complexity on Bob’s side. In particular, the precoding matrix is expressed as

(18) $$W_{\mathrm{ZF}}={\bar{H}}_{b}^{H}{\left({\bar{H}}_{b}{\bar{H}}_{b}^{H}\right)}^{-1}.$$

Subsequently, the transmitted signal is given by

(19) $$sj^{m}=\rho W\mathrm{ZF}xj^{m}+VNn_{0},$$

where $V_{N}$ and $n_{0}$ are analogous to the terms in Equation (11), while ρ is defined as in Equations (12) and (13). The received signal for Bob can then be represented as

(20) $$\begin{array}{cc} y_{b} & =H_{b}s_{j}^{m}+n_{b}\\ \; & =H_{b}\left(\rho W_{\mathrm{ZF}}x_{j}^{m}+V_{N}n_{0}\right)+n_{b}\\ \; & =\rho x_{j}^{m}+n_{b}. \end{array}$$

while the received signal for Eve can be written as

(21) $$\begin{array}{cc} y_{e} & =H_{e}s_{j}^{m}+n_{e}\\ \; & =H_{e}\left(\rho W_{\mathrm{ZF}}x_{j}^{m}+V_{N}n_{0}\right)+n_{e}\\ \; & =\rho H_{e}W_{\mathrm{ZF}}x_{j}^{m}+H_{e}V_{N}n_{0}+n_{e}. \end{array}$$

Under the assumption that $N_{r}=N_{e}$, and with a joint ML detector adopted, the detection process at Bob can be formulated as

(22) $$\left\{\hat{j},{\hat{\alpha }}_{m}\right\}=\arg\min_{\begin{array}{c} 1\leq j\leq N_{r}\\ \alpha_{m}\in A \end{array}}{∥y_{b}-\rho x_{j}^{m}∥}^{2}.$$

Similarly, the corresponding detection rule at Eve is given by

(23) $$\left\{\hat{j},{\hat{\alpha }}_{m}\right\}=\arg\min_{\begin{array}{c} 1\leq j\leq N_{r}\\ \alpha_{m}\in A \end{array}}{∥y_{e}-\rho x_{j}^{m}∥}^{2}.$$

It can be observed from (20) that the injected AN has no effect on Bob’s received signal. By contrast, the term $\rho H_{e}W_{\mathrm{ZF}}$ in (21) is purely real-valued and therefore only modifies the signal amplitude at Eve [16], which may lead to a potential security vulnerability when Eve possesses sufficiently high detection sensitivity. In comparison, the component $H_{e}V_{N}n_{0}$ induces random phase perturbations in Eve’s observation, thereby significantly degrading its detection capability. As a result, the deliberate injection of AN plays a vital role in strengthening the overall transmission security of the proposed scheme.

In this scenario, on the one hand, the receiver Bob is not required to perform near-field channel estimation or compute the precoding matrix. On the other hand, when employing joint ML detection, Bob achieves a significant reduction in computational complexity while preserving optimal performance. However, as digital precoding is adopted on Alice’s side, the associated computational complexity and hardware cost are inevitably higher. The aforementioned two schemes each present distinct advantages and trade-offs. Consequently, Alice and Bob must select and implement the most suitable approach based on the specific operating conditions.

### 3.3. Overall Algorithm

**Figure 3:**
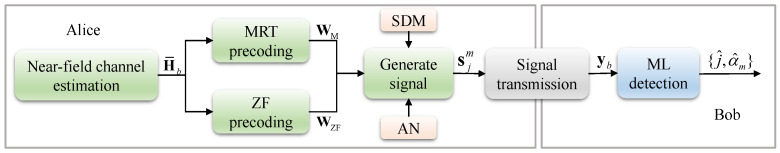
Representation of overall operations with respect to the SDMN-AN system.

The overall operation of the SDMN-AN system is depicted in Figure 3. Both Bob’s and Eve’s receivers are positioned within Alice’s near-field. Initially, Alice performs near-field channel estimation on the Alice–Bob link. Utilizing the estimation results, Alice generates the corresponding precoding matrix. Simultaneously, the transmitted signal is constructed, incorporating the antenna indices, APM symbols, and AN, before being transmitted. Upon reception, Bob employs joint ML detection to estimate the antenna index and demodulate the APM symbol, thereby recovering the transmitted information. Throughout this process, the selection of antenna indices and the incorporation of AN significantly disrupt Eve’s ability to accurately intercept the transmission, thereby reducing its eavesdropping efficacy and ensuring the overall security of the system. Even if Eve applies advanced signal processing, near-field channel mismatch prevents perfect null-space alignment, leading to unavoidable AN leakage in Eve’s received signal.

### 3.4. Complexity Analysis

At the transmitter side, two precoding strategies are considered. For MRT precoding, the precoding matrix is obtained via conjugate channel processing, which requires $𝒪(N_{t}N_{r})$ complex multiplications. In contrast, ZF precoding involves the computation of a matrix inverse of size $N_{r}\times N_{r}$, leading to a computational complexity on the order of $𝒪(N_{r}^{3}+N_{t}N_{r}^{2})$. Therefore, MRT precoding offers lower computational and hardware complexity at the transmitter, while ZF precoding incurs higher processing cost in exchange for simplified receiver-side detection.

At the receiver side, Bob employs joint ML detection over both the receiver index and the modulation symbol. The detection complexity scales with the size of the SDMN-AN symbol set, resulting in a computational complexity of $𝒪(N_{r}M)$ per detection, where *M* denotes the modulation order. This complexity is comparable to that of conventional spatial modulation schemes and remains moderate for small to medium numbers of distributed receivers.

Overall, the proposed SDMN-AN framework provides a flexible trade-off between transmitter-side and receiver-side complexity. MRT-based designs favor low-complexity transmission at the cost of higher detection burden, whereas ZF-based designs shift computational complexity to the transmitter while enabling simplified and efficient detection at the receiver.

## 4. BER Performance Analysis

This section investigates the BER performance of the proposed SDMN-AN scheme. In particular, analytical upper bounds on the BERs experienced by both Bob and Eve are derived under the assumption that a joint ML detector is employed.

Based on the analytical framework developed in [16], the BER upper bound for Bob in the SDMN-AN system can be expressed as

(24) $$P_{B}\leq \frac{1}{\xi 2^{\xi }}\sum_{x_{j}^{m}\in 𝒳}\sum_{\begin{array}{c} x_{k}^{n}\in 𝒳\\ x_{k}^{n}\neq x_{j}^{m} \end{array}}e\left(x_{j}^{m},x_{k}^{n}\right)\;P\left(x_{j}^{m}\to x_{k}^{n}\right),$$

where 𝒳 denotes the set of all possible transmitted symbol vectors under the SDMN-AN scheme. The function $e(x_{j}^{m},x_{k}^{n})$ represents the Hamming distance between the symbol vectors $x_{j}^{m}$ and $x_{k}^{n}$, while $P(x_{j}^{m}\to x_{k}^{n})$ denotes the corresponding pairwise error probability (PEP), which characterizes the probability of erroneously detecting $x_{k}^{n}$ when $x_{j}^{m}$ is transmitted.

In particular, the PEP can be evaluated as

(25) $$\begin{array}{cc} \; & P(x_{j}^{m}\to x_{k}^{n})\\ \; & =P\left\{{∥y_{b}-\rho H_{\mathrm{eff}}x_{j}^{m}∥}^{2}>{∥y_{b}-\rho H_{\mathrm{eff}}x_{k}^{n}∥}^{2}\right\}\\ \; & =P\left.\{R\left\{\rho n_{b}^{H}\left(H_{\mathrm{eff}}x_{k}^{n}-H_{\mathrm{eff}}x_{j}^{m}\right)\right\}>\frac{1}{2}{∥H_{\mathrm{eff}}x_{k}^{n}-H_{\mathrm{eff}}x_{j}^{m}∥}^{2}\right\}, \end{array}$$

where $H_{\mathrm{eff}}$ denotes the effective channel under perfect channel estimation with $H_{\mathrm{eff}}={\bar{H}}_{b}W_{M}$ for MRT precoding and $H_{\mathrm{eff}}=I_{N_{r}}$ for ZF precoding. Moreover, $R\left\{\rho n_{b}^{H}\left(H_{\mathrm{eff}}x_{k}^{n}-H_{\mathrm{eff}}x_{j}^{m}\right)\right\}$ satisfies $R\left\{\rho n_{b}^{H}\left(H_{\mathrm{eff}}x_{k}^{n}-H_{\mathrm{eff}}x_{j}^{m}\right)\right\}∼𝒞𝒩\left(0,\frac{\rho^{2}\sigma_{b}^{2}}{2}{∥H_{\mathrm{eff}}x_{k}^{n}-H_{\mathrm{eff}}x_{j}^{m}∥}^{2}\right)$. Therefore, the PEP can be expressed as

(26) $$P\left(x_{j}^{m}\to x_{k}^{n}\right)=Q\left(\sqrt{\frac{\rho^{2}{∥H_{\mathrm{eff}}x_{k}^{n}-H_{\mathrm{eff}}x_{j}^{m}∥}^{2}}{2\sigma_{b}^{2}}}\right).$$

In a nutshell, the BER upper bound of Bob can be derived using Equation (24) in conjunction with Equation (26).

Subsequently, similar to BER upper bound of Bob, BER upper bound of Eve can be given by

(27) PE≤1ξ2ξ∑xjm∈𝒳∑xkn∈𝒳xkn≠xjme(xjm,xkn)P′(xjm→xkn),

where $P^{\prime }\left(x_{j}^{m}\to x_{k}^{n}\right)$ is the PEP corresponding to Eve’s estimated channel ${\bar{H}}_{e}$, which can be derived as

(28) $$\begin{array}{cc} \; & P^{\prime }\left(x_{j}^{m}\to x_{k}^{n}\right)\\ \; & =P\left\{{∥y_{e}-\rho {H^{\prime }}_{\mathrm{eff}}x_{j}^{m}∥}^{2}>{∥y_{e}-\rho {H^{\prime }}_{\mathrm{eff}}x_{k}^{n}∥}^{2}\right\}\\ \; & =P\left\{{∥\rho {H^{\prime }}_{\mathrm{eff}}x_{j}^{m}+H_{e}V_{N}n_{0}+n_{e}-\rho {H^{\prime }}_{\mathrm{eff}}x_{j}^{m}∥}^{2}\right.\\ \; & \;\;\left.>{∥\rho {H^{\prime }}_{\mathrm{eff}}x_{k}^{n}+H_{e}V_{N}n_{0}+n_{e}-\rho {H^{\prime }}_{\mathrm{eff}}x_{k}^{n}∥}^{2}\right\}\\ \; & =P\left\{ℜ\;\left\{\rho {\left(H_{e}V_{N}n_{0}\right)}^{H}\left(x_{k}^{n}-x_{j}^{m}\right)+\rho n_{e}^{H}\left(x_{k}^{n}-x_{j}^{m}\right)\right\}\right.\\ \; & \;\;\left.>\frac{\rho^{2}}{2}({∥{H^{\prime }}_{\mathrm{eff}}x_{j}^{m}-x_{k}^{n}∥}^{2}-{∥{H^{\prime }}_{\mathrm{eff}}x_{j}^{m}-x_{j}^{m}∥}^{2})\right\}. \end{array}$$

where ${H^{\prime }}_{\mathrm{eff}}$ denotes the effective channel under perfect channel estimation with ${H^{\prime }}_{\mathrm{eff}}={\bar{H}}_{e}W_{M}$ for MRT precoding and ${H^{\prime }}_{\mathrm{eff}}={\bar{H}}_{e}W_{\mathrm{ZF}}$ for ZF precoding. Moreover, it can be derived that $\rho {\left(H_{e}V_{N}n_{0}\right)}^{H}\left(x_{k}^{n}-x_{j}^{m}\right)∼𝒞𝒩\left(0,\frac{\rho^{2}\sigma_{b}^{2}}{2}{∥{\left(H_{e}V_{N}\right)}^{H}\left(x_{k}^{n}-x_{j}^{m}\right)∥}^{2}\right)$ and $\rho n_{e}^{H}\left(x_{k}^{n}-x_{j}^{m}\right)∼𝒞𝒩\left(0,\frac{\rho^{2}\sigma_{e}^{2}}{2}{∥x_{k}^{n}-x_{j}^{m}∥}^{2}\right)$. Therefore, the PEP $P^{\prime }\left(x_{j}^{m}\to x_{k}^{n}\right)$ can be formulated as

(29) $$P^{\prime }\left(x_{j}^{m}\to x_{k}^{n}\right)=Q\left(\frac{\rho {∥{H^{\prime }}_{\mathrm{eff}}x_{j}^{m}-x_{k}^{n}∥}^{2}-\rho {∥{H^{\prime }}_{\mathrm{eff}}x_{j}^{m}-x_{j}^{m}∥}^{2}}{\sqrt{2\sigma_{b}^{2}{∥{\left(H_{e}V_{N}\right)}^{H}\left(x_{k}^{n}-x_{j}^{m}\right)∥}^{2}+2\sigma_{e}^{2}{∥x_{k}^{n}-x_{j}^{m}∥}^{2}}}\right).$$

Hence, the BER upper bound of Eve can be derived by combining Equations (27) and (29).

It is worth noting that, unlike the far-field case, the near-field channel exhibits distance-dependent phase curvature across the transmit array, which causes Eve’s received signal components to experience severe phase misalignment even under perfect channel estimation. Combined with the residual AN leakage induced by near-field channel mismatch, this effect results in significant amplitude and phase uncertainty at Eve, thereby fundamentally degrading its detection performance.

## 5. Simulation Results

In this section, the security performance of the proposed SDMN-AN scheme is evaluated by comparing its BER with that of the DM-AN scheme presented in [19]. Unless otherwise specified, the comparison is conducted over a line-of-sight (LoS)-dominated near-field channel under the assumption of perfect channel estimation. For both theoretical and Monte Carlo simulation results, the following parameters were used: $N_{t}=256$, $N_{r}=2$, $N_{e}=1$, and $D=\lambda_{c}/2$. The positions of Bob and Eve are provided in Table 1. The quadrature phase shift keying (QPSK) scheme was employed, and the BER for each simulation was averaged over $10^{6}$ realizations. For convenience, it is assumed that the noise power is identical at both Bob and Eve, i.e., $\sigma_{b}^{2}=\sigma_{e}^{2}=\frac{1}{\mathrm{SNR}}$, where SNR denotes the signal-to-noise ratio.

**Figure 4:**
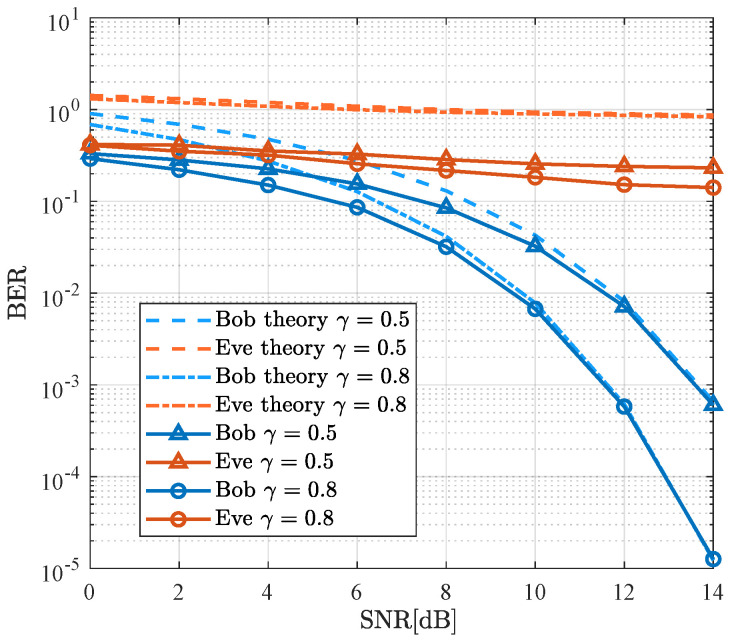
Theoretical and simulated BER performance of the proposed SDMN-AN scheme with ZF precoding and different γ.

Figure 4 presents the performance comparison of both theoretical and simulated BER for Bob and Eve, utilizing the SDMN-AN method with ZF precoding under various values of γ. The modulation scheme is QPSK with a spectral efficiency of 3 bits/Hz/s. Analysis reveals that as the SNR increases, both the theoretical and simulated BER for Bob gradually decrease, with the simulated BER converging to the theoretical value. Conversely, the BER for Eve remains elevated. Specifically, for Bob, the case with γ=0.8 offers a 2 dB improvement over the case with γ=0.5 at a BER level of $10^{-3}$. Meanwhile, variations in γ have minimal impact on the BER for Eve. The change in γ alters the distribution of power between the precoded signal and AN. A larger γ leads to more power being allocated to the precoded signal, thereby improving Bob’s performance. However, the presence of AN ensures that Eve’s performance remains poor, demonstrating the security of the SDMN-AN scheme, even when Eve is in close proximity to Bob.

**Figure 5:**
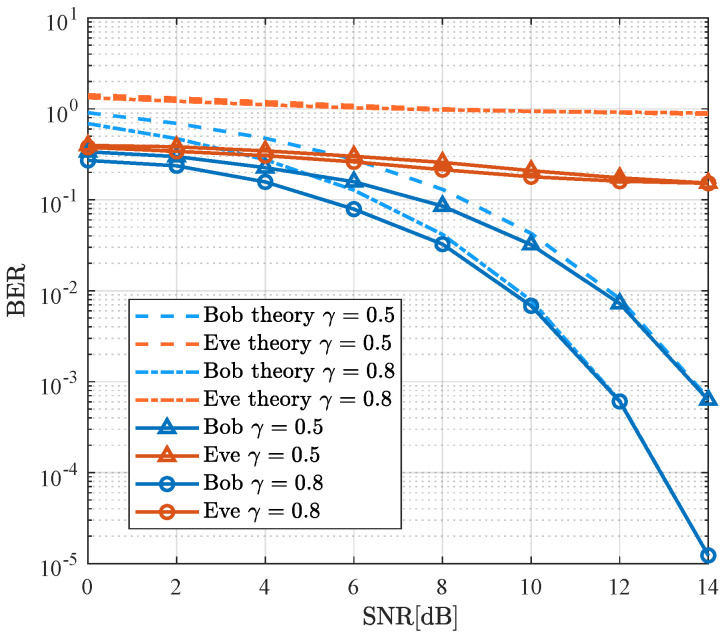
Theoretical and simulated BER performance of the proposed SDMN-AN scheme with MRT precoding and different γ.

Figure 5 illustrates the theoretical and simulated BER performance for both Bob and Eve using the SDMN-AN method with MRT precoding across various values of γ. Similar to the results presented in Figure 4, the SNR increase results in a decrease in both theoretical and simulated BER for Bob, with the simulated BER approaching the theoretical BER. Notably, when γ=0.8, a 2 dB performance gain is observed for Bob compared to the γ=0.5 case at a BER level of $10^{-3}$. Due to the use of AN, the BER of Eve remains high, with minimal impact from changes in γ. Furthermore, MRT precoding offers the advantage of reducing Alice’s hardware requirements. These results confirm that by modulating γ, the SDMN-AN scheme can achieve varied security performance, even with Eve positioned closely to Bob.

**Figure 6:**
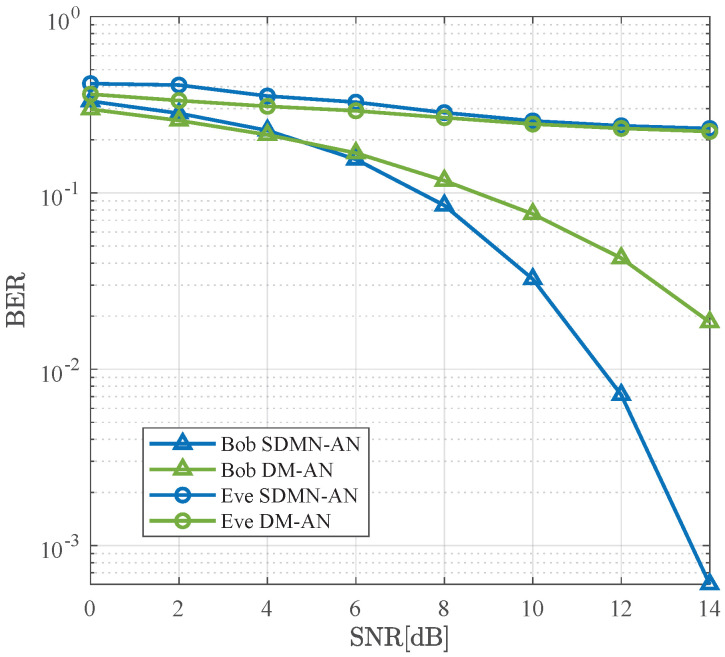
BER performance comparison between the proposed SDMN-AN scheme with ZF precoding and DM-AN scheme when γ=0.5.

Figure 6 compares the BER performance of the SDMN-AN and DM-AN schemes using ZF precoding at γ=0.5. The modulation scheme for SDMN-AN is QPSK, while DM-AN uses 8PSK, with both schemes having the same spectral efficiency of 3 bits/Hz/s. As the SNR increases, the BER performance for both schemes improves for Bob, but Eve’s performance remains poor. Particularly, when the BER is $10^{-2}$, Bob’s performance using SDMN-AN method exhibits a 3 dB improvement over DM-AN method. This is attributed to the fact that SDMN-AN encodes some bits into receiver indices, which enhances the accuracy of joint ML detection. Conversely, DM-AN encodes all bits into constellation points, thus increasing the demodulation complexity. These results validate that SDMN-AN not only enhances spectral efficiency but also improves Bob’s performance while maintaining security.

**Figure 7:**
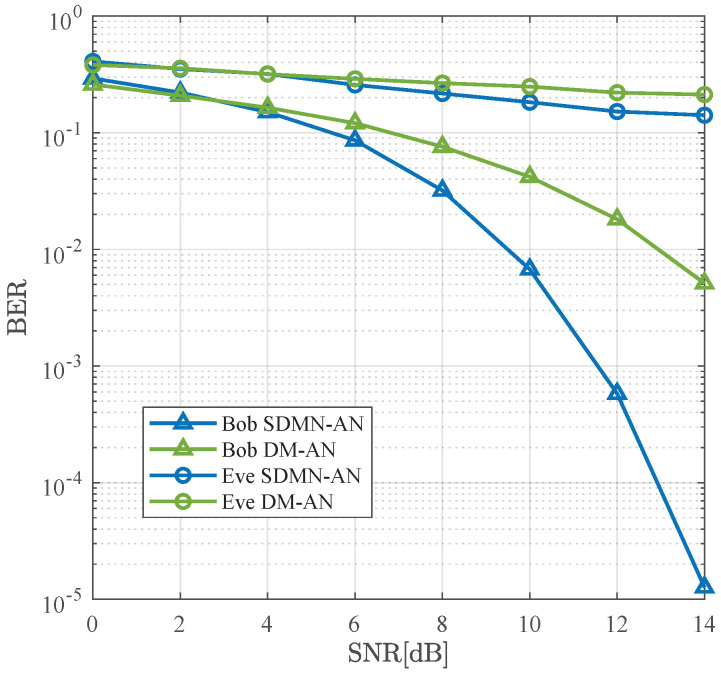
BER performance comparison between the proposed SDMN-AN scheme with ZF precoding and DM-AN scheme when γ=0.8.

As illustrated in Figure 7, it compares the BER performance between SDMN-AN and DM-AN schemes using ZF precoding at γ=0.8. The modulation scheme for SDMN-AN is QPSK, while DM-AN utilizes 8PSK, and both schemes maintain the same spectral efficiency. Although different modulation orders are used, joint ML detection is applied to both schemes to ensure a fair comparison under the same detection criterion. Similar to Figure 6, as the SNR increases, the BER for both schemes decreases for Bob, with Eve’s performance remaining poor. However, at a BER of $10^{-2}$, SDMN-AN outperforms DM-AN by 3.5 dB gains. This improvement is attributable not only to the modulation of bits into receiver indices but also to the increased power allocated to the precoded signal as γ increases, further enhancing Bob’s performance. These results show that increasing γ improves both spectral efficiency and security, further protecting against Eve’s eavesdropping.

**Figure 8:**
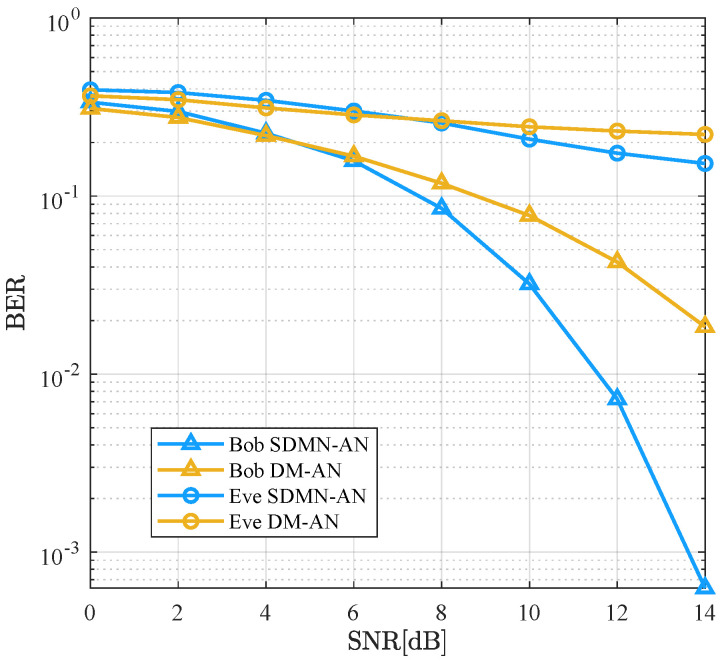
BER performance comparison between the proposed SDMN-AN scheme with MRT precoding and DM-AN scheme when γ=0.5.

Figure 8 compares the BER performance of SDMN-AN and DM-AN schemes using MRT precoding at γ=0.5. The modulation scheme for SDMN-AN is QPSK, while DM-AN employs 8PSK, with both schemes having the same spectral efficiency. Compared to ZF precoding, MRT precoding reduces Alice’s hardware overhead but increases the detection complexity at Bob’s side. In this case, the SDMN-AN scheme demonstrates good performance. Specifically, for a BER level of $10^{-2}$, SDMN-AN outperforms DM-AN by 3 dB gains, confirming that MRT precoding still ensures system security and spectral efficiency while reducing hardware overhead.

**Figure 9:**
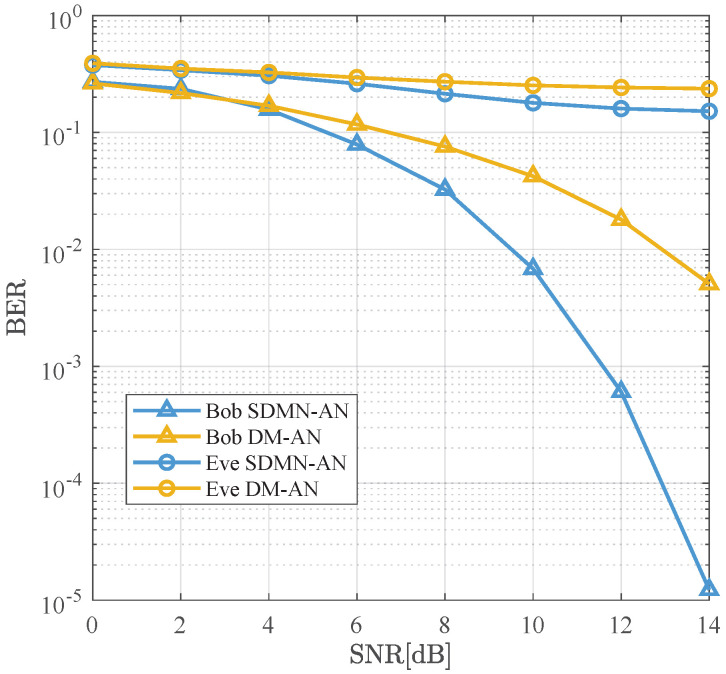
BER performance comparison between the proposed SDMN-AN scheme with MRT precoding and DM-AN scheme when γ=0.8.

As shown in Figure 9, it presents the BER comparison of SDMN-AN and DM-AN schemes using MRT precoding at γ=0.8. The modulation for SDMN-AN is QPSK, while for DM-AN, it is 8PSK. Notably, when Bob’s BER is $10^{-2}$, SDMN-AN method achieves a 3.5 dB improvement over DM-AN method, surpassing the performance seen at γ=0.5. This performance gain is attributed to the increased power allocated to the precoded signal. Both schemes effectively prevent Eve’s successful reception and demodulation, ensuring robust security against eavesdropping.

**Figure 10:**
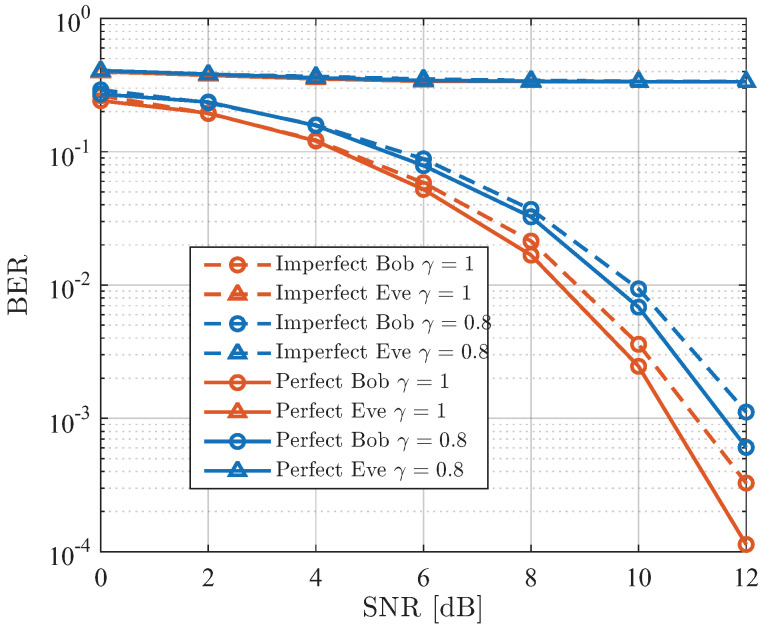
BER comparison using MRT precoding between perfect and imperfect channel estimation for the proposed SDMN-AN scheme.

Figure 10 illustrates the BER performance of the proposed SDMN-AN scheme using MRT precoding under imperfect channel estimation for different values of γ. The channel estimation error is modeled as a phase perturbation of $10^{\circ }$, which is incorporated as a multiplicative factor $\exp\left(j\theta_{\mathrm{error}}\pi /180\right)$ with $\theta_{\mathrm{error}}=10^{\circ }$. It is observed that, in the presence of channel estimation errors, the BER performance of Bob degrades compared to the perfect channel estimation case. However, the performance loss remains within an acceptable range. Meanwhile, Eve consistently experiences a significantly degraded BER performance, thereby ensuring the communication security of the proposed scheme.

**Figure 11:**
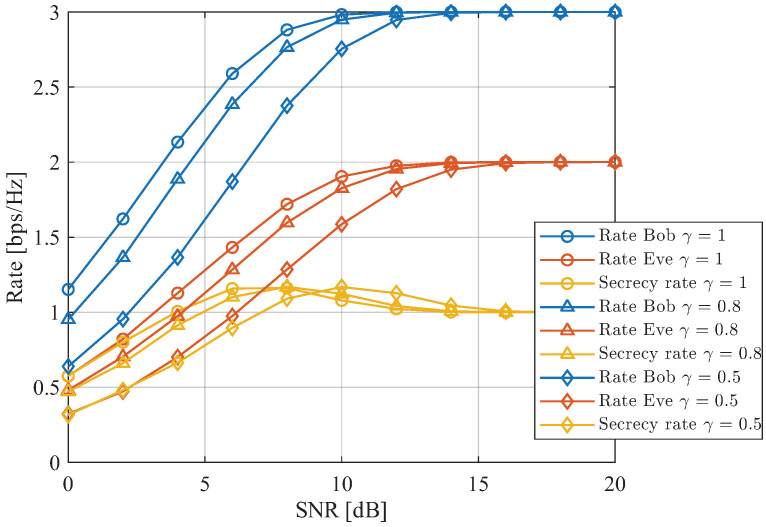
Rate performance comparison of Bob, Eve, and the resulting secrecy rate under different γ values in the proposed SDMN-AN scheme.

Figure 11 depicts the rate performance of the proposed SDMN-AN scheme for different numbers of receive antennas $N_{r}$ and values of γ. As shown in Figure 11, the achievable rate and secrecy rate vary with different choices of γ. Specifically, as γ decreases, the SNR at which the secrecy rate attains its peak shifts to a higher value. In practical implementations, the parameter γ can be properly selected according to system requirements to achieve the desired secrecy performance. These results further indicate that the secrecy advantage of the proposed SDMN-AN scheme is not solely attributed to power allocation or modulation order, but fundamentally arises from the near-field propagation characteristics, which enable distance-dependent signal discrimination and persistent AN leakage at the eavesdropper.

**Figure 12:**
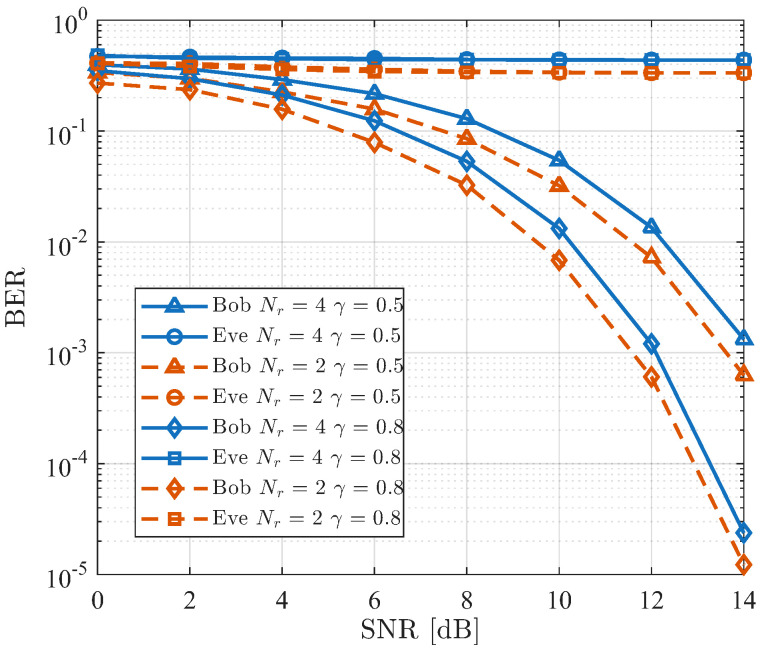
BER comparison using MRT precoding between different $N_{r}$ for the proposed SDMN-AN scheme.

Figure 12 shows the BER performance of the proposed SDMN-AN scheme using MRT precoding under different numbers of receive antennas $N_{r}$ and values of γ. The locations are specified in Table 2 for $N_{r}=4$. It can be observed that, as $N_{r}$ increases, although the spectral efficiency is improved, the BER performance of the intended receiver degrades. Moreover, for a given $N_{r}$, the BER performance improves with increasing γ. In contrast, Eve consistently suffers from severe interference, thereby maintaining the communication security of the proposed scheme. In addition, a smaller value of γ corresponds to a higher proportion of AN, which, although slightly degrades Bob’s BER performance, further suppresses Eve’s interception capability, highlighting the practical trade-off between reliability and security.

## 6. Conclusions

This paper introduced the SDMN-AN system, a novel approach for securing near-field communications by leveraging multi-point cooperative receivers and AN to mitigate eavesdropping risks. By integrating indices of Bob with modulation symbols, SDMN-AN significantly improved spectral efficiency. The incorporation of AN further impaired the ability of Eve to successfully reconstruct the transmitted signal. Two precoding strategies—MRT with joint ML detection and ZF precoding with joint ML detection—were utilized, offering a balanced trade-off between security and computational efficiency. The simulation results demonstrated that the SDMN-AN system enhances communication reliability for Bob while restricting Eve’s ability to intercept the transmitted signal. Compared to conventional DM-AN systems, SDMN-AN not only improves security but also optimizes spectral efficiency, making it a promising solution for secure near-field communications. In addition, the proposed SDMN-AN framework is well suited for application scenarios such as cooperative sensor networks, short-range secure sensing, and indoor near-field communication systems, where distributed receivers naturally arise and near-field propagation dominates. In these settings, the ability to exploit receiver indices and AN provides an effective and practical means for enhancing physical-layer security.

## Figures and Tables

**Table 1:** Locations of Bob and Eve.

	Steering Angles (^∘^)	Distance (m)
Bob	{70, 110}	{15, 30}
Eve	{73}	{15}

**Table 2:** Locations of Bob and Eve for $N_{r}=4$.

	Steering Angles (^∘^)	Distance (m)
Bob	{35, 70, 110, 155}	{10, 15, 30, 20}
Eve	{73}	{15}

## Data Availability

The data presented in this study are available on request from the corresponding author.
